# Year Books of Medical Progress

**Published:** 1884-11

**Authors:** 


**Year Books of Medical Progress:** A Year Book of Surgery for 1883. Edited by CHARLES H. KNIGHT, M. D. A Year Book of Therapeutics for 1883. Edited by ROYAL W. AMIDON, M. D. G. P. Putnam's Sons, 27 and 29 West Twenty-third Street, New York; 25 Henrietta Street, Covent Garden, London. 1884.

The high standing of the publishers of these works gives assurance of their worth and value and of their professional excellence. The object is to condense within a moderate space the most important contributions to surgery found in the current literature of the year, and also the advances in therapeutics in the same period. The progress in surgery has been most marked, and the volume devoted to this subject gives a most valuable resumé, without endeavoring to approach completeness in so vast a field. The advances of this department in thyroidectomy, in supra-pubic lithotomy, in the digital exploration of the bladder, syphilology, etc., are well portrayed in the work devoted to surgery. The progress in therapeutics is not a flattering one, according to our author. He says: "There has been little of genuine value added to our knowledge by workers in the field of experimental therapeutics. The enterprise of wholesale druggists and manufacturers exceeds the labors of the therapeutist in the number and variety of drugs and medicinal preparations thrown upon the market." For the profession, as well as the public, these works are valuable. They present a condensed statement of the more important advances made in the respective departments of which they treat. We think the publishers have conferred upon the profession important aids in their labors, and we know of no works in which are condensed so much information as in these.

